# Pulpotec® Beyond Pulpotomy: A Case Series of Versatile Applications in Vital Pulp Therapy Procedures in Permanent Teeth

**DOI:** 10.7759/cureus.115566

**Published:** 2026-08-31

**Authors:** Sushmita Pattnaik, Varsha Sharma, Manisha Kusuma, Sameer Chauhan, Priyanka Shah, Kanika Singh Dhull, Pratik Surana

**Affiliations:** 1 Pediatric and Preventive Dentistry, Government Medical College and Hospital, Sundargarh, IND; 2 Pediatric and Preventive Dentistry, Department of Health and Family Welfare - Government of Odisha, Bhubaneswar, IND; 3 Pediatric Dentistry, Maharshi Vashishtha Autonomous State Medical College, Rampur, IND; 4 Prosthodontics, Lakewood Family Dental, Bloomington, USA; 5 Prosthodontics, K. M. Shah Dental College and Hospital, Sumandeep Vidyapeeth (Deemed to be University), Vadodara, IND; 6 Pedodontics and Preventive Dentistry, AI Badar Rural Dental College and Hospital, Gulbarga, IND; 7 Pediatric Dentistry, Kalinga Institute of Dental Sciences, Bhubaneswar, IND; 8 Pedodontics and Preventive Dentistry, Maitri College of Dentistry and Research Centre, Durg, IND

**Keywords:** dental pulp diseases, endodontics, permanent teeth, pulp capping, pulpotomy, vital pulp therapy

## Abstract

Vital pulp therapy is a conservative approach aimed at preserving pulp vitality in teeth affected by deep caries or pulp exposure. Pulpotec® has been widely used as a pulpotomy medicament in primary teeth; however, its application in permanent teeth remains limited. This case series illustrates the use of Pulpotec® in five permanent molars that underwent indirect pulp capping, direct pulp capping, and pulpotomy. Clinical and radiographic evaluations were performed over a follow-up period of up to six months. All treated teeth remained asymptomatic with no tenderness, swelling, or signs of periapical pathology. Radiographic findings showed favorable outcomes, including radiographic hard-tissue formation, canal narrowing, and maintenance of periodontal ligament space. Within the limitations of this study, Pulpotec® demonstrated promising results as a pulp-preserving medicament in permanent teeth.

## Introduction

Pulpitis resulting from deep carious lesions has traditionally been managed with conventional endodontic therapy, involving the complete removal of pulp tissue followed by root canal treatment. Although effective in eliminating infection and relieving symptoms, this approach results in loss of pulp vitality and its biological functions. In recent years, increasing emphasis has been placed on conservative and biologically based management of pulpitis, aiming to preserve the vitality of the dental pulp whenever possible. This shift aligns with the principles of minimally invasive dentistry. It has led to renewed interest in vital pulp therapy as a viable alternative to conventional root canal treatment in appropriately selected cases [1].

Vital pulp therapy comprises procedures such as indirect pulp capping, direct pulp capping, and pulpotomy, which aim to maintain pulp vitality and promote the healing of the remaining healthy pulp tissue. Traditionally, materials such as calcium hydroxide have been widely used in these procedures for their antibacterial properties and ability to stimulate radiographic hard-tissue formation [2]. However, limitations such as poor sealing ability and the formation of tunnel defects in dentin bridges have prompted the development of newer bioactive materials. More recently, mineral trioxide aggregate, Biodentine, and other calcium silicate-based materials have shown improved clinical outcomes due to their superior sealing ability, biocompatibility, and ability to stimulate dentin regeneration [3-5].

Pulpotec® is a radiopaque pulp medicament containing polyoxymethylene, iodoform, and dexamethasone, which provide antimicrobial and anti-inflammatory properties. It has been widely used as a pulpotomy agent, particularly in primary teeth, with several studies reporting favorable clinical and radiographic success rates. However, despite its documented effectiveness in the primary dentition, evidence for its use in vital pulp therapy in the permanent dentition remains limited, particularly beyond conventional pulpotomy. Its proposed biological action differs from that of contemporary calcium silicate-based materials. Rather than being considered a bioactive regenerative material, Pulpotec® has been described as eliciting a localized fixation and cicatrization response at the pulp-medicament interface, intended to maintain the underlying pulpal tissue [6-8]. Dexamethasone contributes primarily through its anti-inflammatory activity. Although Pulpotec® has been investigated extensively in the primary dentition, evidence regarding its use in vital pulp therapy in the permanent dentition remains limited.

Therefore, the present case series aimed to describe the clinical and radiographic outcomes associated with the use of Pulpotec® in different vital pulp therapy procedures, including indirect pulp capping, direct pulp capping, and pulpotomy in permanent molars. The study also aimed to document radiographic pulpal responses observed during short-term follow-up.

## Case presentation

Study design

The present study was designed as a clinical case series demonstrating the application of Pulpotec® in various vital pulp therapy procedures in permanent teeth. The study was conducted and reported in accordance with the CARE (Case Report) guidelines for clinical case reporting.

Case selection

A total of five cases with deep carious lesions in permanent molars were included in the study. Patients were evaluated through detailed clinical and radiographic examination prior to treatment. Only teeth with signs suggestive of a vital pulp and an absence of periapical pathology were considered suitable for vital pulp therapy procedures.

The inclusion criteria comprised teeth with deep carious lesions that approximated or involved the pulp, with a vital pulp that responded positively to pulp sensibility testing, and had no clinical signs of swelling, sinus tract formation, or abnormal mobility. Radiographically, the teeth had no evidence of periapical pathology and had to be considered restorable with the possibility of achieving an adequate coronal seal. The exclusion criteria comprised non-vital teeth or teeth with necrotic pulp, teeth demonstrating radiographic evidence of periapical radiolucency, teeth with severe periodontal involvement or mobility, and patients with systemic conditions that could adversely affect healing.

Diagnostic procedure

Diagnosis was established based on the patient's history, clinical examination, pulpal sensibility testing, and radiographic findings. Clinical assessment included evaluation of the characteristics, duration, and provocation of pain, as well as percussion and palpation tests. Pulpal sensibility was assessed using thermal testing and electric pulp testing, where feasible. Based on the clinical and sensibility findings, a standardized pulpal diagnosis was assigned. The apical diagnosis was established based on percussion, palpation, clinical examination, and radiographic findings. Intraoral periapical radiographs were obtained to assess the extent of caries, pulp involvement, root development, and periapical status.

Treatment protocol

All procedures were performed under local anesthesia with appropriate isolation. Caries removal was performed using sterile round burs in a slow-speed handpiece under copious water irrigation. Depending on the clinical condition of the pulp and the extent of caries, different vital pulp therapy procedures were performed. For indirect pulp capping, infected dentin was selectively removed while retaining a thin layer of affected dentin overlying the pulp to avoid pulp exposure. For direct pulp capping, the pulp medicament was placed directly over a small pulp exposure after achieving hemostasis. In cases requiring pulpotomy, the inflamed coronal pulp was removed while preserving the healthy radicular pulp. In all cases, Pulpotec® paste (Produits Dentaires SA, Switzerland) was placed as the pulp medicament. The teeth were then restored with definitive materials, such as composite resin or Cention N®, to ensure an adequate coronal seal.

Follow-up evaluation

Patients were recalled periodically for clinical and radiographic evaluation, with follow-up periods ranging from one to six months depending on the individual case. Clinical success was defined by the absence of spontaneous pain, tenderness to percussion or palpation, swelling, and sinus tract formation, along with a normal functional response. Radiographic assessment included evaluation for periapical or furcal radiolucency, changes in the periodontal ligament space, restoration integrity, and observable changes in the pulp space or mineralized tissue. Radiographic hard-tissue formation/barrier and canal narrowing, when observed, were recorded as pulpal responses. In particular, radiographic evidence of pulp canal narrowing or obliteration was not interpreted as definitive evidence of maintained pulp vitality. The absence of clinical symptoms and pathological radiographic changes during the available follow-up period was considered indicative of a favorable short-term clinical and radiographic outcome.

Ethical considerations

This case series was conducted in accordance with the ethical standards of the institutional research committee and the Declaration of Helsinki. Formal ethical approval was not required for this case series, as it involved routine clinical procedures. Written informed consent was obtained from all patients/parents prior to treatment and for publication of clinical data and radiographic images.

Case series presentation

Case 1: Indirect Pulp Capping in Permanent Molar

An 11-year-old male patient presented with sensitivity in the upper right posterior region associated with deep caries in tooth 16. Clinical examination revealed a deep occlusal carious lesion (Figure 1A). The tooth responded positively to pulp sensibility testing, with no abnormal response to percussion or palpation. Radiographic examination revealed deep caries approximating the pulp chamber without periapical pathology (Figure 1B). The tooth was diagnosed as having reversible pulpitis and normal apical tissues. After local anesthesia was administered and the area was isolated, the infected dentin was selectively removed (Figure 1C). Pulpotec® was placed as the pulp medicament over the remaining dentin, followed by a composite resin restoration (Figure 1D). At the six-month follow-up, the tooth remained asymptomatic with no tenderness to percussion. Radiographic examination revealed the absence of periapical pathology, with evidence of hard-tissue deposition/barrier, apparent narrowing of the pulp chamber, and a radiopaque calcific focus (Figure 1E).

**Figure 1:**
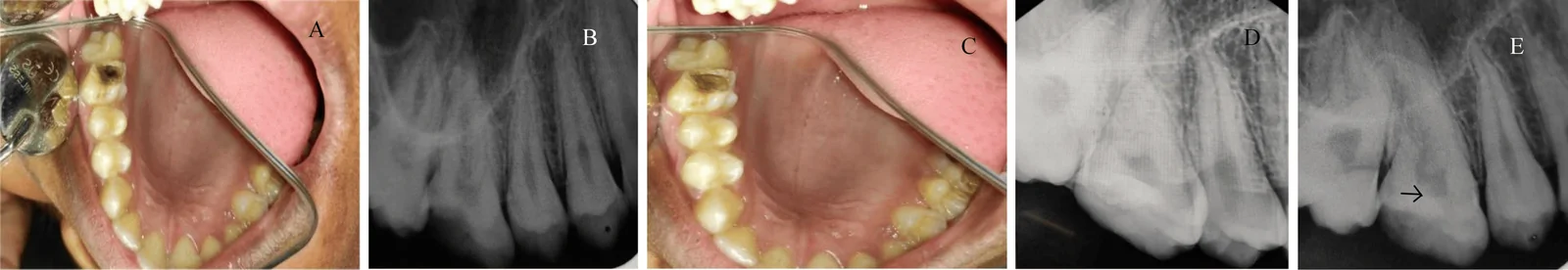
Case 1 (A) Preoperative clinical photograph showing a deep occlusal carious lesion in tooth 16. (B) Preoperative intraoral periapical radiograph showing deep caries approximating the pulp chamber without periapical pathology. (C) Intraoperative photograph demonstrating selective caries removal prior to placement of Pulpotec®. (D) Immediate postoperative photograph showing placement of Pulpotec® and restoration of the cavity with resin composite. (E) Follow-up radiograph at six months demonstrating absence of periapical pathology and apparent recession of the pulp chamber, suggestive of radiographic hard-tissue deposition (arrow).

Case 2: Indirect Pulp Capping in Permanent Molar

A 12-year-old male patient presented with a chief complaint of food lodgment and occasional sensitivity in the upper left posterior region. Clinical examination revealed a deep occlusal carious lesion involving tooth 26. The tooth responded positively to pulp sensibility testing, with no abnormal response to percussion or palpation. Radiographic examination revealed a radiolucency involving the enamel and dentin and approaching the pulp chamber, without evidence of periapical pathology (Figure 2A). Based on the clinical and diagnostic findings, the tooth was diagnosed with reversible pulpitis and normal apical tissues. Under local anesthesia and appropriate isolation, selective caries removal was performed, preserving a thin layer of affected dentin over the pulp to avoid exposure of the pulp (Figure 2B). Pulpotec® paste was placed over the deepest portion of the cavity, followed by a protective base and definitive restoration. The immediate postoperative radiograph confirmed adequate placement of the restorative material (Figure 2C). At the six-month follow-up, the patient remained asymptomatic. Radiographic evaluation demonstrated apparent narrowing/partial obliteration of the pulp space. This finding was recorded as a calcific pulpal response and was not considered, in isolation, evidence of maintained pulp vitality (Figure 2D).

**Figure 2:**
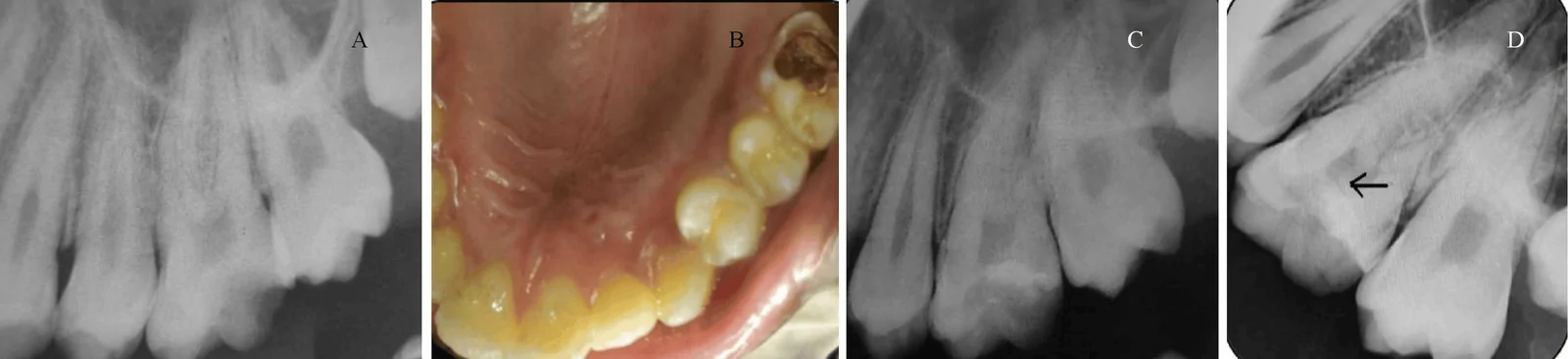
Case 2 (A) Preoperative intraoral radiograph showing a radiolucency involving the enamel and dentin and approaching the pulp chamber in tooth 26. (B) Selective caries removal. (C) Postoperative radiograph. (D) Six-month follow-up showing radiographic hard tissue formation/barrier (arrow).

Case 3: Direct Pulp Capping in Permanent Molar

A 14-year-old female patient presented with a deep occlusal carious lesion in tooth 46. Radiographic examination revealed deep caries extending to the pulp chamber, without evidence of periapical pathology (Figure 3A). The tooth responded positively to pulp sensibility testing, with no abnormal response to percussion or palpation. The tooth was diagnosed with reversible pulpitis and normal apical tissues. During caries excavation, a small exposure of the pulp occurred. Hemostasis was achieved using a sterile cotton pellet moistened with saline. Pulpotec® paste was placed directly over the exposed pulp tissue, followed by placement of a protective base and restoration with Cention N®. The immediate postoperative radiograph confirmed adequate restoration and medicament placement (Figure 3B). At the six-month follow-up, the tooth remained asymptomatic with no signs of tenderness or swelling. Radiographic evaluation showed an intact restoration and absence of periapical pathology, supporting a favorable short-term clinical and radiographic outcome (Figure 3C).

**Figure 3:**
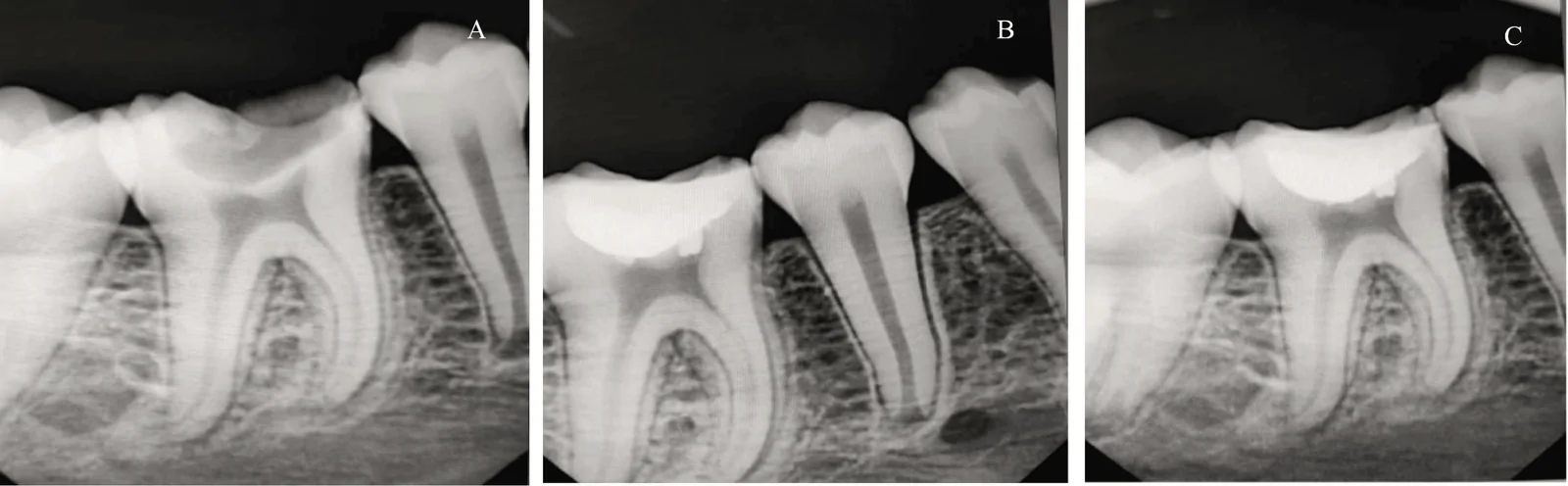
Case 3 (A) Preoperative intraoral periapical radiograph showing deep occlusal caries in tooth 46 approximating the pulp. (B) Immediate postoperative radiograph after placement of Pulpotec® over the pulp exposure site and restoration with Cention N®. (C) Follow-up radiograph at six months demonstrating intact restoration and absence of periapical pathology.

Case 4: Direct Pulp Capping in an Immature Permanent Molar

A 13-year-old male patient presented with deep caries involving tooth 37. Radiographic examination revealed a deep carious lesion with immature root formation and open apices (Figure 4A). The tooth responded positively to pulp sensibility testing, with no abnormal response to percussion or palpation. Based on the clinical and diagnostic findings, the tooth was diagnosed with reversible pulpitis and normal apical tissues. Following caries excavation, a small pulp exposure was encountered. After achieving hemostasis, Pulpotec® paste was placed directly over the exposed pulp tissue and sealed with a protective base. A definitive restoration was then placed. The immediate postoperative radiograph confirmed the placement of the pulp medicament and restoration (Figure 4B). At the six-month follow-up, the tooth remained asymptomatic with normal function. Radiographic examination showed intact restoration and absence of periapical pathology, indicating a favorable short-term clinical and radiographic outcome (Figure 4C).

**Figure 4:**
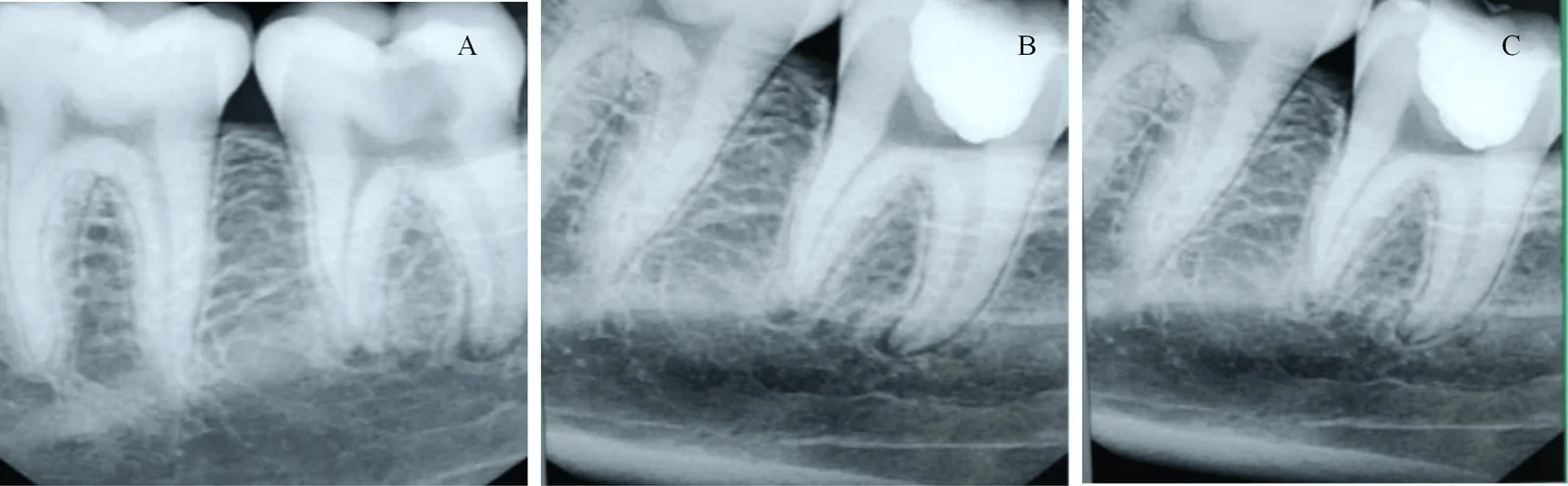
Case 4 (A) Preoperative radiograph showing a deep carious lesion in tooth 37 with immature root formation. (B) Immediate postoperative radiograph following direct pulp capping with Pulpotec®. (C) Six-month follow-up radiograph demonstrating intact restoration and absence of periapical pathology.

Case 5: Pulpotomy in Permanent Molar

A 10-year-old female patient presented with deep caries involving tooth 46, with clinical findings suggestive of pulpal involvement. Radiographic examination revealed a deep carious lesion extending into the pulp chamber (Figure 5A). The tooth responded positively to pulp sensibility testing, with no abnormal response to percussion or palpation. Based on the clinical and diagnostic findings, the tooth was diagnosed with symptomatic irreversible pulpitis with normal apical tissues. Under local anesthesia and isolation, the coronal pulp tissue was removed to perform a pulpotomy. Hemostasis was achieved, and Pulpotec® paste was placed over the radicular pulp stumps. A protective base was applied, followed by definitive restoration. The immediate postoperative radiograph confirmed placement of the medicament (Figure 5B). At the six-month follow-up, the tooth remained clinically asymptomatic. Radiographic evaluation demonstrated a preserved periodontal ligament space and the absence of periapical pathology, indicating a favorable treatment outcome (Figure 5C).

**Figure 5:**
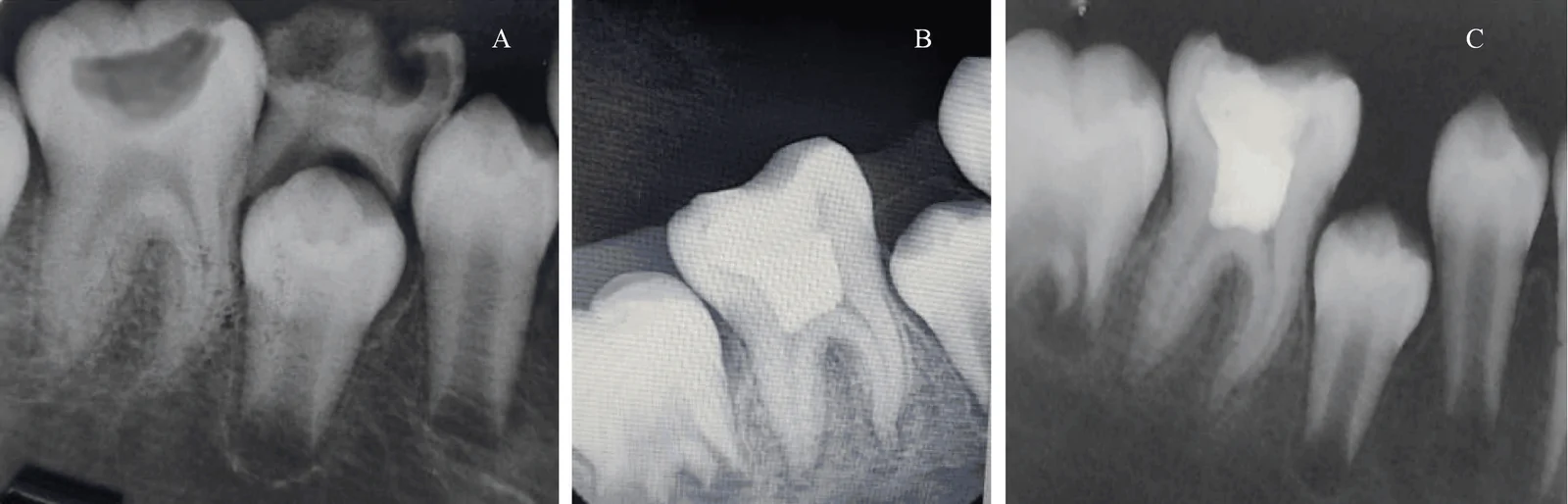
Case 5 (A) Preoperative radiograph showing a deep carious lesion approaching the pulp in 46. (B) Immediate postoperative radiograph showing pulpotomy with Pulpotec®. (C) Six-month follow-up radiograph showing an intact restoration and no pathology.

## Discussion

Vital pulp therapy has gained increasing acceptance as a conservative approach for managing teeth with deep carious lesions and pulp exposure. Traditionally, such cases were treated with complete pulpectomy and root canal therapy; however, recent evidence suggests that inflamed pulp tissue retains significant regenerative potential when bacterial contamination is controlled and an adequate coronal seal is achieved [9].

Indirect pulp capping aims to preserve pulp vitality by selectively removing infected dentin while avoiding exposure of the pulp. In the present case series, cases treated with indirect pulp capping demonstrated favorable outcomes, including radiographic hard-tissue formation and canal narrowing at the six-month follow-up. These findings are consistent with previous studies reporting successful maintenance of pulp vitality following selective caries removal [10].

Direct pulp capping is indicated in appropriately selected cases with limited pulp exposure, where favorable outcomes depend on controlling contamination and hemostasis, ensuring the biocompatibility of the pulp-covering material, and providing an effective coronal seal. In the present series, teeth managed with direct pulp capping remained asymptomatic and showed no radiographic evidence of periapical pathology during the available follow-up period. These findings indicate favorable short-term clinical and radiographic outcomes; however, the absence of symptoms or periapical changes does not establish histological healing or confirm the formation of reparative dentin [11].

In immature permanent teeth, preservation of pulp vitality is critical for continued root development and apical closure. The favorable outcome observed in the immature tooth case highlights the regenerative capacity of the pulp tissue when treated with appropriate vital pulp therapy procedures [12]. Longer-term follow-up would be required to determine whether treatment results in continued root maturation and apical development.

Pulpotomy, traditionally limited to primary or immature permanent teeth, has recently been explored as a treatment option in mature permanent teeth. The favorable short-term outcome observed in the present case supports the concept that pulpotomy can serve as a conservative alternative to conventional root canal treatment in selected cases [13,14].

Pulpotec® contains components with antimicrobial, anti-inflammatory, and tissue-fixative properties. Its proposed mechanism differs from that of contemporary calcium silicate-based materials. Histological investigations in primary teeth have described a localized cicatricial/fixative response at the pulp-medicament interface, with preservation of tissue deeper within the pulp, rather than a regenerative process comparable to that associated with bioactive calcium silicate cements. Dexamethasone may reduce inflammatory activity through its corticosteroid-mediated anti-inflammatory effects; however, a reduction in inflammation or symptoms should not be equated with regeneration or definitive pulpal healing. In the present case series, the favorable clinical findings therefore support only the possibility of short-term pulpal preservation/stabilization and do not establish a specific biological mechanism. Previous studies evaluating Pulpotec® in primary teeth have reported favorable clinical and radiographic outcomes [6,8]. While most of the literature supports its use in the primary dentition, the present case series demonstrates its potential applicability across multiple vital pulp therapy procedures in the permanent dentition.

The favorable outcomes observed may be attributed to appropriate case selection, maintenance of aseptic conditions, effective control of pulpal bleeding, and placement of a well-sealed coronal restoration. These factors are critical determinants of success in vital pulp therapy [15].

However, the present study has limitations, including a small sample size and short follow-up period. Further studies with larger sample sizes and long-term follow-up are required to validate the long-term effectiveness of Pulpotec® in permanent teeth.

## Conclusions

Within the limitations of this case series, Pulpotec® demonstrated favorable clinical and radiographic outcomes across different vital pulp therapy procedures in permanent teeth, including indirect pulp capping, direct pulp capping, and pulpotomy. Teeth treated with indirect pulp capping showed radiographic hard-tissue deposition and narrowing or partial obliteration of the pulp space, representing a favorable radiographic pulpal response. Cases managed with direct pulp capping remained clinically asymptomatic, with intact restorations and no periapical pathology during follow-up. Similarly, the pulpotomy case exhibited maintenance of periodontal ligament space and absence of radiographic signs of pathology, indicating a favorable short-term clinical and radiographic outcome. These findings suggest that Pulpotec® may be considered a potential pulp medicament for the conservative management of pulpitis in selected cases involving permanent teeth. However, further studies with larger sample sizes and longer follow-up are required to validate its long-term effectiveness.
